# A multicenter randomized placebo-controlled trial of intravenous thyroxine for heart-eligible brain-dead organ donors

**DOI:** 10.1186/s13063-021-05797-2

**Published:** 2021-11-27

**Authors:** Rajat Dhar, Dean Klinkenberg, Gary Marklin

**Affiliations:** 1 Department of Neurology, Washington University in St. Louis School of Medicine, St. Louis, MO USA; 2 Mid-America Transplant, St. Louis, MO USA

**Keywords:** Brain death, Organ donation, Heart transplant, Thyroid hormone, Donor management

## Abstract

**Background:**

Brain death frequently induces hemodynamic instability and cardiac stunning. Impairments in cardiac performance are major contributors to hearts from otherwise eligible organ donors not being transplanted. Deficiencies in pituitary hormones (including thyroid-stimulating hormone) may contribute to hemodynamic instability, and replacement of thyroid hormone has been proposed as a means of improving stability and increasing hearts available for transplantation. Intravenous thyroxine is commonly used in donor management. However, small controlled trials have not been able to demonstrate efficacy.

**Methods:**

This multicenter study will involve organ procurement organizations (OPOs) across the country. A total of 800 heart-eligible brain-dead organ donors who require vasopressor support will be randomly assigned to intravenous thyroxine for at least 12 h or saline placebo. The primary study hypotheses are that thyroxine treatment will result in a higher proportion of hearts transplanted and that these hearts will have non-inferior function to hearts not treated with thyroxine. Additional outcome measures are the time to achieve hemodynamic stability (weaning off vasopressors) and improvement in cardiac ejection fraction on echocardiography.

**Discussion:**

This will be the largest randomized controlled study to evaluate the efficacy of thyroid hormone treatment in organ donor management. By collaborating across multiple OPOs, it will be able to enroll an adequate number of donors and be powered to definitively answer the critical question of whether intravenous thyroxine treatment increases hearts transplanted and/or provides hemodynamic benefits for donor management.

**Trial registration:**

ClinicalTrials.govNCT04415658. Registered on June 4, 2020

**Supplementary Information:**

The online version contains supplementary material available at 10.1186/s13063-021-05797-2.

## Administrative information

Note: the numbers in curly brackets in this protocol refer to SPIRIT checklist item numbers. The order of the items has been modified to group similar items (see http://www.equator-network.org/reporting-guidelines/spirit-2013-statement-defining-standard-protocol-items-for-clinical-trials/).

**Table:** 

Title {1}	A Multicenter Randomized Placebo-Controlled Trial of Intravenous Thyroxine for Heart-Eligible Brain-Dead Organ Donors
Trial registration {2a and 2b}.	Clinicaltrials.gov NCT04415658. Registered June 4, 2020. https://clinicaltrials.gov/ct2/show/NCT04415658
Protocol version {3}	3.0; 11.01.2021
Funding {4}	None (internal administrative support provided by Mid-America Transplant)
Author details {5a}	^1^Department of Neurology, Washington University in St. Louis School of Medicine, St. Louis, MO. ^2^Mid-America Transplant, St. Louis, MO.
Name and contact information for the trial sponsor {5b}	Mid-America Transplant 1110 Highlands Plaza Drive East, #100 Saint Louis, MO 63110
Role of sponsor {5c}	The sponsor has no role in the design or analysis of the trial data. They will not have any role in the writing of the manuscript or the decision to submit the report for publication.

## Introduction

### Background and rationale {6a}

Brain death frequently induces hemodynamic instability and cardiac stunning [1, 2]. Impairments in cardiac performance are major contributors to hearts from otherwise eligible organ donors not being transplanted [3]. Deficiencies in pituitary hormones (including thyroid-stimulating hormone) may contribute to hemodynamic instability [4, 5]. Replacement of thyroid hormone has been proposed as a means of improving stability and increasing hearts available for transplantation [6]. Intravenous thyroxine is now commonly used in donor management across the USA, either for all donors or only those with hemodynamic instability or those who are being considered for heart donation [7, 8]. Large retrospective studies have found associations between the use of hormonal resuscitation, including thyroid hormone, and more hearts transplanted [7, 9]. However, several small controlled trials have not been able to confirm the efficacy of thyroid hormone in improving heart function after brain death or increasing the chances of hearts being transplanted [10, 11].

We recently performed two small parallel single-center randomized studies of thyroid hormone in heart-eligible brain-dead (BD) organ donors. The first evaluated whether intravenous triiodothyronine (T3) was superior to thyroxine (T4) in hemodynamically unstable BD donors with cardiac stunning [12]. This was based on the rationale (supported by some experimental data) that T3 is the active hormone and its levels decline more rapidly after brain death [13]. We were able to measure cardiac performance (left ventricular ejection fraction, LVEF) both immediately prior to starting infusion of T3 or T4 and immediately after 8 h of therapy. This physiologic proof-of-principle study demonstrated that LVEF improved comparably in both groups over this short time frame (from 38–45% to 50–53%) and both groups could be progressively weaned off vasoactive agents prior to organ recovery. Furthermore, after adjusting for group imbalances (likely due to small study size), hearts were transplanted in a similar proportion of the T3 and T4 groups (rate of heart utilization was 43% in this population). This study did not support the superiority of T3 (a more expensive intervention) over T4 in the management of unstable BD heart donors.

Our next study aimed to test whether T4 infusion would be superior to placebo in heart-eligible donors with reduced LVEF despite being off vasopressors. Both studies aimed to enroll and start infusion within 12 h of BD (at least defined by time when declaration occurred). Neither studies were blinded but evaluation of LVEF was performed blind to treatment allocation. Median improvement in LVEF was 10% with T4 compared with 5% without thyroid hormone (*p* = 0.24), although intention-to-treat efficacy analysis was limited by the fact that several of those randomized to T4 either did not receive or had this intervention discontinued prematurely due to emergent hypertension or tachycardia [14]. There was a trend to more hearts being transplanted in the T4 group (59 vs. 27%, *p* = 0.14) as well as more organs in total transplanted per donor (median of 5 vs. 3, *p* = 0.009). Although this study suggested that thyroid hormone could be efficacious in increasing hearts transplanted, definitive conclusions were precluded by its small sample size (a consequence of enrolling at only one center) and group imbalances despite randomization. Therefore, we believe that there remains scientific equipoise and significant interest among the organ procurement organization (OPO) and transplant communities in determining whether thyroid hormone does actually improve heart function and result in more hearts transplanted.

In fact, scientific research to determine the optimal interventions for donor management has been receiving increasing interest [15]. To date, most studies have been retrospective and not adequately controlled [16, 17]. Recently, OPOs have begun collaborating more intently to answer important donor management questions. A council focused on research was formed as part of the *Association of Organ Procurement Organizations* (AOPO). This Organ Donation Research Council (ODRC) aims to bring together OPOs and interested parties (such as transplant physicians and scientists) to advance the science of organ and tissue donation. Collaborations nurtured as part of ODRC led to a multicenter randomized study that demonstrated that naloxone, frequently used (based on retrospective studies) to improve lung function, did not actually improve oxygenation or result in more lungs transplanted than a saline placebo [18]. A few other randomized donor management studies have been performed [19–21].

## Objectives {7}

The primary hypothesis of this study is that intravenous thyroxine (T4) will increase the proportion of hearts transplanted when given early after brain death to heart-eligible hemodynamically unstable potential organ donors. The primary safety hypothesis is that hearts transplanted from donors receiving T4 will have non-inferior graft survival to those from the placebo group at 30 days. Secondary objectives are to evaluate whether T4 will reduce time to wean off vasopressors and improve cardiac ejection fraction.

## Trial design {8}

This is a multicenter prospective randomized trial. Randomization will occur in parallel groups to intravenous thyroxine or saline placebo in a 1:1 ratio. Randomization will be in blocks, stratified by OPO site, allowing each site to serve as its own control (given heterogeneity in donor management protocols between OPOs). The administration of study drug or placebo will not be blinded, given logistical limitations at donor hospitals and OPO facilities where this pragmatic study is being conducted.

## Methods: participants, interventions, and outcomes

### Study setting {9}

This study will be performed by various organ procurement organizations (OPOs) across the country (full list available on clinicaltrials.gov, with the current list of OPOs provided in Additional file 2). Participants will be patients already declared dead by neurologic criteria (i.e., brain dead) and who have provided authorization (first-person or by living surrogates) for both organ donation *and* for research. They may be physically located either/both in the hospitals in which they were declared dead and/or at an independent recovery/donor management facility of the OPO. The donor component has been deemed not to involve human subjects’ research, and participating hospitals and OPOs are therefore not engaged in human subjects’ research. The coordinating site (Washington University in St. Louis) will be the research site responsible for collecting recipient outcome data. A waiver of consent has been obtained for de-identified collection of this human recipient data.

### Eligibility criteria {10}

Inclusion criteria:

1. Declared dead by neurologic criteria
2. Provided authorization for organ donation *and* for research
3. Donor age of 14–55 years (inclusive) *and* weight ≥ 45 kg (100 lbs)
4. On one or more vasopressor and/or inotropes (not including vasopressin)

Exclusion criteria:

1. Brain death declared > 24 h prior
2. Known CAD or MI (by history, EKG, or previous cardiac catheterization)*
3. Significant valvular heart disease (by history of echo)*
4. Prior sternotomy or cardiac surgery*
5. Donor at VA hospital
6. Received intravenous or oral thyroid hormone in the past month
7. Known HIV+ status
8. Other reasons preventing the donor from receiving study drug (determined by the on-site coordinator)
  *Sufficient to exclude donor heart from being considered for transplantation

### Who will take informed consent? {26a}

Donors are dead and their surrogates have all provided authorization for research prior to screening for this study. This will be verified by organ procurement coordinators who are managing the donor management process and screening for study eligibility. Recipients of organs from those enrolled in this study are human subjects, but we have obtained a waiver of consent to collect de-identified graft function/outcome data on those receiving hearts from donors enrolled in this trial.

### Additional consent provisions for collection and use of participant data and biological specimens {26b}

All data utilized for analysis will be collected as part of donor care including standard UNOS and Scientific Registry of Transplant Recipients (SRTR) data variables. No additional biologic specimens will be collected.

## Interventions

### Explanation for the choice of comparators {6b}

Intravenous thyroxine (T4) is commonly employed at almost all OPOs around the country as part of standard hormonal resuscitation, either for all donors or only those who are hemodynamically unstable [8]. It is recommended (without level I evidence) in several donor management guidelines [22]. It is usually given as an infusion with or without an initial bolus (protocols vary). A pilot randomized comparison of thyroid hormone formulations (T3 vs. T4) did not suggest any important benefits of T3 over T4 [12]. Treatment with T4 will be compared to no thyroid hormone (during the first 12 h, open-label T4 may be used in both groups after this period, at the discretion of the OPO medical director or transplant team).

### Intervention description {11a}

Study infusion (saline or thyroxine) will be commenced as soon as possible after randomization. Serum-free T4 and TSH levels will be drawn prior to giving the drug. The T4 will be prepared by mixing 500 μg of drug in 500 ml of normal saline (i.e., concentration of 1 μg/ml) and enclosing the bag in an opaque sleeve. The placebo will be a 500-ml bag of normal saline (without active drug) also enclosed in an opaque sleeve. The infusion from this bag will be started at 30 ml/h (corresponding to 30 μg/h) and run for at least 12 h unless adverse effects occur and the infusion is stopped prematurely (see titration protocol below). Vasoactive medications will be weaned as expeditiously as possible. When the donor is considered hemodynamically stable, initial transthoracic echocardiography (TTE) will be ordered and obtained as soon as possible. Repeat serum-free T4 level will be drawn prior to organ recovery in both groups. Organ allocation will proceed per Organ Procurement and Transplantation Network (OPTN) policy and routine allocation practices. All other aspects of donor management will also follow local OPO protocols.

### Criteria for discontinuing or modifying allocated interventions {11b}

T4 infusion can be titrated down in the following circumstances, per study protocol:

- Systolic blood pressure above 180 mm Hg *and* increase of 30 mm Hg above baseline
- Heart rate over 120 bpm *and* increase by 20 bpm above baseline
- New tachyarrhythmia (atrial fibrillation, atrial flutter, supraventricular tachycardia, ventricular tachycardia) *or* new-onset ectopy (ventricular premature contractions > 6/min)
- Other significant changes, at discretion of the coordinator

The dose should be reduced in increments of 10 μg/h (i.e., to 20 then 10 μg/h). The infusion can be discontinued if hemodynamic instability persists despite weaning. Open-label T4 can be given to the placebo group after 12 h at the discretion of the managing team (whether for persistent hemodynamic instability, low ejection fraction on echo, or physician preference). T4 can also be continued beyond 12 h of study infusion for the same reasons in the intervention group.

### Strategies to improve adherence to interventions {11c}

There will be a 1-month run-in period at each site where screening logs will be checked by the site and central study coordinators for completeness and accuracy. They will review all cases where donors were not enrolled within 24 h or if other reasons for ineligibility were provided and provide feedback. The site and study coordinators will have weekly conference calls for the first month (and then monthly thereafter) to discuss issues with adherence. They will also provide feedback to individual OPO personnel if study protocols are violated. The study statistician will perform monthly audits of study data for completeness.

### Relevant concomitant care permitted or prohibited during the trial {11d}

All standard OPO donor management practices will be continued throughout the study period until and including organ allocation and recovery. The only intervention prohibited is the administration of thyroid hormone to those donors allocated to the placebo group for the first 12 h after randomization.

### Provisions for post-trial care {30}

Data collection on non-human organ donors ends at the time of organ procurement or end of donor management (for authorized brain-dead potential donors who do not go on to donate organs).

## Outcomes {12}

The primary outcome for this study is the proportion of hearts transplanted (the number of hearts transplanted divided by the total number of potential donors—including those authorized but not recovered).

The primary safety outcome for this study is 30-day graft survival in those receiving hearts from donors enrolled in this study. This is defined by patient survival with the originally transplanted heart and no mechanical circulatory support. It will be assessed primarily at 30 days but also at 1 year.

Secondary outcomes will include:

1. Time from randomization to weaning off vasopressors: defined as being off all vasoactive agents, except for vasopressin (≤ 1 unit per hour will not be considered on vasopressors as this is routinely used for management of diabetes insipidus)
2. Proportion weaned off vasopressors by 12 h (same criteria as #1)
3. Vasopressor-inotrope score at 12 h (excluding vasopressin ≤1 unit/h)
4. Time achieving hemodynamic stability adequate to order first echocardiography
5. Ejection fraction measured on first donor echocardiography
6. Lungs and total thoracic organs transplanted
7. Total number of organs transplanted
8. Additional outcomes routinely collected from heart transplant recipients will also be aggregated and analyzed, including the need for post-transplant mechanical circulatory support and other measures of primary graft dysfunction

### Safety endpoints

The following adverse events will be prospectively collected on all study donors. All events deemed either related or potentially related to the study drug infusion will be reviewed by the site coordinator. Any serious related events (defined as hemodynamic instability leading to cardiac arrest or donor loss prior to organ recovery) will be forwarded to the central study coordinators and safety monitoring board for review:

1. Severe hypertension (systolic BP > 200 mm Hg)
2. Tachycardia (HR > 150 and increased more than 20 over baseline)
3. New/worsened tachyarrhythmia (SVT, atrial fibrillation, ventricular tachycardia, or fibrillation)
4. Cardiac arrest or any donor loss prior to the OR
5. Fever—new, above 102°C
6. New skin rash

### Participant timeline {13}

Table 1.

**Table 1:**
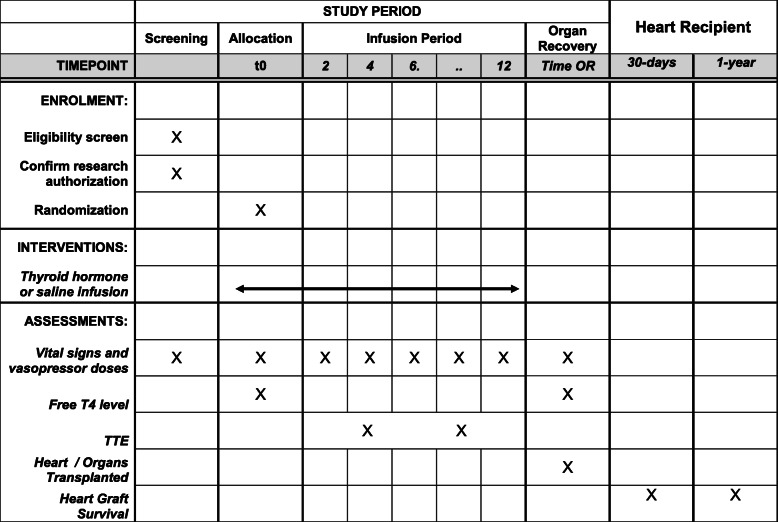
Participant timeline {13}

## Sample size {14}

The proportion of potential heart donors who were able to provide a heart for transplantation in our pilot studies was between 30 and 60% [12, 14], a rate concordant with the recent literature [23]. Proposed effect sizes of thyroid hormone treatment have ranged as high as an absolute increase of 20% or more in proportion of hearts transplanted in non-randomized studies [24]; a large retrospective analysis of UNOS data found that those who received thyroid hormone (57% of 40,124 donors) had more hearts transplanted [7]. The increase was from 25.8% without treatment to 35% with thyroid hormone (among all donors, not only those who were strictly heart-eligible, as required in this study). Based on our pilot data and this literature, we propose an absolute treatment effect size of 10% as the threshold for this study. In order to detect an increase in the proportion of hearts transplanted from 35 to 45%, we will need 752 total donors (376 per group), assuming 80% power and a one-sided alpha of 0.025. Because we expect some loss of donors after enrollment (e.g., clinical issues that interrupt adherence to the protocol), we will enroll a total of 800 donors (400 in each study group).

For our safety analysis in recipients of donor hearts, we have calculated a sample size necessary to demonstrate non-inferiority in graft survival. Based on recent data provided by SRTR, the expected 30-day graft survival after heart transplantation is 96%. We will test for a decrease of no greater than 6% in graft survival (i.e., from 96 to 90%; see the “36” section for a full explanation of assumptions and testing). With a one-sided alpha of 0.025, we would need 336 recipients (168 per group) to have 80% power to exclude such a difference. Therefore, based on our sample size of 800 donors and an expectation that overall at least 40% of donor hearts will be transplanted, we expect a minimum of 320 recipients for this analysis; this sample size will provide a power of 78% for non-inferiority. It is likely that more than 40% of hearts will be transplanted; in fact, we saw in excess of 50% of hearts transplanted in our pilot data. Therefore, we expect to have more than the required number of recipients to achieve 80% power. This sample size of recipients would also provide 80% power to demonstrate non-inferiority at the 1-year survival endpoint.

## Recruitment {15}

In our pilot data, approximately 30% of all donors were eligible for this study. The number of total donors managed at OPOs varies based on their donor service area, but ranges from 60 to 300 per year (mean of 160 donors per year, in our preliminary site survey). This will mean that between 20 and 100 donors will be eligible at each site per year (depending on size) with an expected average of 40–50 enrolled per site. Therefore, we are recruiting at least 15 OPO sites in order to complete recruitment within an 18-month study period. We currently have 14 sites involved in the study with 12 actively recruiting (see Additional file 2 for list).

## Assignment of interventions: allocation

### Sequence generation {16a}

A random sequence of 1 s and 0 s will be computer-generated by the central coordinating site in blocks of 30 for each participating OPO site (i.e., stratified by site) to designate the study and placebo groups. A Web-accessible randomization sheet will be provided to each site.

### Concealment mechanism {16b}

Only once eligibility has been confirmed for a given donor will the on-site organ procurement coordinator login to the website with the randomization list and determine (using the next available row) to which group the donor has been assigned. The coordinator will enter the UNOS# of the donor on the randomization form, as well as the time and date of enrollment in the study before communicating the study assignment to OPO staff. The site study coordinator will review the randomization log to ensure that all eligible donors are being randomized and that sequential randomization rows are utilized.

### Implementation {16c}

Randomization sequence of T4 vs. saline will be generated by the central coordinating statistician. Participants will be enrolled by the organ procurement coordinators at each site managing the donor after determining eligibility. They will determine the allocation to one of the study groups and assign participants to their randomized intervention.

## Assignment of interventions: blinding

### Who will be blinded {17a}

Care providers will not be blinded to study groups. Organ donors (being deceased) will be unaware of treatment. Assessment of cardiac function on echocardiography will also be blinded to the study group.

### Procedure for unblinding if needed {17b}

The study will not be blinded to coordinators or to those performing organ allocation (e.g., transplant teams). Furthermore, we will ensure that it is clearly documented in DonorNet (the platform accessible to transplant teams that contains all relevant donor-related data) that each participant is enrolled in this study and to which group they were randomized. Each participant can also receive open-label T4 at the termination of the 12-h study period, at the discretion of the OPO or transplant teams.

## Data collection and management

### Plans for assessment and collection of outcomes {18a}

Each OPO will identify a site study coordinator who will be responsible for ensuring the proper completion of data collection forms. The central coordinating OPO will create the data collection forms and distribute them to each study coordinator (data collection forms shown in Additional file 1). The central coordinating site will train each study coordinator on data collection and data entry procedures. Recipient data will be collected by the central site by matching donor UNOS IDs to the SRTR database of recipient and graft outcomes. No identifiable data on recipients will be collected and this data will not be shared with the sites.

### Plans to promote participant retention and complete follow-up {18b}

No participants will be lost to follow-up as the final assessment occurs at organ recovery, which is available for all donors. However, it is possible that some donors may have incomplete data or may not finish the protocol once it is started. For example, a donor may be authorized for donation but may not become an organ donor (“authorized, not recovered” status). Such a donor does not undergo organ recovery and so will not have data at the final endpoint. However, we will analyze groups as randomized, i.e., by intention-to-treat, imputing these non-recovered donors as not having hearts transplanted. Similarly, we will analyze all those randomized to T4 within that study group even if they do not receive the drug or have it discontinued before 12 h.

### Data management {19}

Study-specific data will be entered onto a paper data flowsheet with the UNOS ID for each participant and the assigned group marked at the top. This data sheet will be forwarded to the site study coordinator who will enter participant data into a secure REDCap database. This has built-in data validation and range checks. This study data will be supplemented by matching each enrolled donor (by UNOS ID) to their SRTR demographic data. This includes donor age, sex, and other important variables such as cause of death and comorbidities. The central coordinating statistician will perform data quality checks monthly and will work with the study coordinators at each OPO to resolve any quality issues.

### Confidentiality {27}

No participant data will be shared outside of the study except when UNOS IDs are provided to the OPTN/SRTR to acquire data on whether transplanted hearts functioned and recipients survived. No recipient identifying data will be collected.

### Plans for collection, laboratory evaluation, and storage of biological specimens for genetic or molecular analysis in this trial/future use {33}

No biologic specimens will be collected for this trial or for future use.

## Statistical methods

### Statistical methods for primary and secondary outcomes {20a}

We will present descriptive statistics for demographic and clinical variables, including by study group, but will not perform statistical comparisons of differences between study groups for baseline variables that should be balanced by randomization. We will first evaluate the primary outcome, the proportion of hearts transplanted, presenting the absolute and relative differences between the study groups, along with 97.5% one-sided confidence intervals. For our primary analysis, we will test the association between treatment and proportion of hearts transplanted, using a one-sided chi-square test at a significant level of 0.025. Then, we will proceed to a multivariate model that adjusts for potentially confounding variables and random site effect. The SRTR registry has developed comprehensive organ-specific risk-adjustment models [25]. Older age and blood group are consistently the most important variables impacting whether hearts are transplanted. For this reason, we will include age and blood type as relevant prespecified covariates. Since randomization was stratified by site, and imbalance in sample size across sites is expected, we will also include the site as a random effect in our multivariate model. This will provide subject-specific measures of treatment effect, in terms of a conditional effect size and adjusted odds ratio (with 97.5% one bound confidence interval).

Given that a systematic assessment of non-randomized studies using T4 for heart donors has suggested an improved rate of hearts transplanted, while prior small randomized studies have suggested no effect (i.e., no studies suggesting that T4 therapy results in worse heart outcomes) [10], we have selected a superiority design as most appropriate for our primary outcome. We are seeking to ascertain whether the addition of T4 infusion to standard donor care results in more hearts transplanted. Therefore, our null hypothesis is that T4 treatment results in an equal or lower rate of hearts transplanted, meaning our alternate hypothesis is that T4 treatment is superior to no treatment. If the null hypothesis were not excluded, there would be no rationale to use T4 in addition to standard donor management and no benefit to focusing on a two-sided test evaluating whether it was worse than placebo. Therefore, statistical tests will be one-tailed at a stringent significance level of 0.025. We will also present the effect size with confidence intervals for all outcomes. The analysis will follow an intention-to-treat model. However, we will also perform a secondary per-protocol analysis, including only those receiving at least 6 h of study infusion. We will also analyze only those in the placebo group who did not receive open-label T4, for a secondary analysis.

We will analyze the primary safety endpoint of recipient graft survival using a non-inferiority method. Our baseline assumption, based on 5-year data (from 2016 to 2020) provided by SRTR, is that 30-day graft survival for hearts will be 96%. We assume this baseline rate for both study groups, as there is no evidence to suggest that graft survival is lower in recipients of hearts from donors administered thyroid hormone. In fact, a recent study suggested treatment was associated with improved graft survival [23]. The null hypothesis is that heart graft survival is lower in the T4 treated group by at least a clinically significant margin (set at 6%) compared with that observed in hearts transplanted from the placebo donors. This non-inferiority margin was selected with input of the data and safety monitoring committee (DSMC), including a thoracic transplant surgeon and ethicist. It aligns with that used in other trials assessing graft function after heart transplantation which used similar baseline assumptions (96% graft survival) but allowed more liberal margins (10%) [26]. We believe that setting the lower limit of tolerance for this safety analysis at 90% graft survival is most appropriate. We will construct two-sided 95% confidence intervals for the difference in graft survival between the groups, allowing us to test for one-sided non-inferiority at alpha 0.025. This will be performed in the intention-to-treat cohort (i.e., all hearts from enrolled donors, by randomized study group) but we will also evaluate non-inferiority using a per-protocol methodology (i.e., evaluating outcomes in recipients who received hearts from donors who received at least 6 h of T4 infusion compared to those who did not).

Our plans for analyzing the secondary outcomes are as follows: for outcome measures that are time-to-event variables such as time to get off vasopressors and time to order first echo, we will perform a Cox regression model to assess hazard ratio (95% confidence interval) between groups. We will calculate the vasopressor-inotrope score, VIS [27] as: VIS = dopamine dose (μg/kg/min) + dobutamine dose (μg/kg/min) + 100 × epinephrine dose (μg/kg/min) + 100 × norepinephrine dose (μg/kg/min) + 10 × milrinone dose (μg/kg/min) + 100 × phenylephrine dose (μg/kg/min) + 10,000 × vasopressin dose (U/kg/min—only for doses > 1 U/h). We will compare the decrease in VIS from the start of thyroid hormone or saline infusion until 12 h (end of study infusion), using analysis of covariate method with predictor variables that include the baseline score and the treatment groups and adjusting for site effect. Cardiac ejection fraction will be evaluated using a linear mixed model adjusting for random site effect. If normality assumption is violated, data transformation will be performed. Count data regarding thoracic and total organs transplanted will be compared using Generalized Estimating Equations, with the study group as the independent variable and expected organs transplanted as a covariate. All secondary outcome analyses will be a two-tailed test at a significance level of 0.05.

### Interim analyses {21b}

The DSMC statistician will perform one interim analysis when 376 donors have been enrolled with evaluable data. The trial may be terminated early if, based on the interim findings, there is either superiority or clear inferiority of T4 treatment compared to placebo (at a threshold of 0.01). The trial may also be terminated if there is inferiority of graft outcomes in either group, observed at 30 days in those hearts transplanted and observed for this period. The DSMC will provide recommendations to the study PIs, who will make final decisions about whether to stop or continue the trial.

### Methods for additional analyses (e.g., subgroup analyses) {20b}

We will analyze the primary outcome (proportion of hearts donated) for each OPO individually. Sample sizes will vary between OPOs, so we may only have power to detect large effect sizes. Still, these OPO-specific analyses could provide valuable insights about trends at each OPO. We will also perform prespecified subgroup analyses of (1) donors with time to start infusion < 12 h vs. > 12 h from BD determination; (2) LVEF result (normal, > 50%, abnormal ≤50%).

### Methods in analysis to handle protocol non-adherence and any statistical methods to handle missing data {20c}

Donors who are not managed according to the protocol (e.g., not managed properly on T4) will be included in the initial analyses following an intent-to-treat model. Subsequent analyses may exclude those cases in order to evaluate the impact of the protocol on donors who completed it successfully.

### Plans to give access to the full protocol, participant-level data, and statistical code {31c}

The full protocol is available on clinicaltrials.gov. The participant-level (donor) dataset will be made available to study investigators and their respective OPOs. It may be provided to other OPOs, on request. No recipient outcome data will be shared.

## Oversight and monitoring

### Composition of the coordinating center and trial steering committee {5d}

The coordinating center is comprised of the study PIs and the central study statistician. The coordinating center will be responsible for oversight of the study. The study statistician will review data entered by study sites for completeness and perform periodic checks for accuracy. The central study coordinator will ensure transfer of additional donor-related data and demographics for each enrolled donor to the central database on a quarterly basis. The respective study OPOs will provide donor data only and not be involved in collecting or analyzing recipient data.

### Composition of the data monitoring committee, its role, and reporting structure {21a}

The data and safety monitoring committee (DSMC) is composed of several experienced but independent transplant researchers covering a wide range of expertise. It includes a transplant physician and researcher, a thoracic transplant surgeon, two epidemiologists/statisticians experienced with donor and transplant research, a medical ethicist, and a medical director of an OPO not involved in the study. The DSMC statistician will analyze the primary outcome and primary safety data at the time of the interim analysis. These results will be shared with the entire DSMC, who will discuss and provide a recommendation on whether to continue recruitment or whether to terminate the study. The DSMC members are independent from the study investigators and are not involved in the procedures of the study other than this review and the review of any serious safety events.

### Adverse event reporting and harms {22}

Study data forms include prospective ascertainment of adverse events entered by the organ procurement coordinators caring for each donor (events listed under “Safety endpoints” section in the “18” section). Any events deemed either related or potentially related to the study drug infusion will be reviewed by the site coordinator. The site coordinator will report all AEs that are unanticipated and/or serious to the central site, within 72 h, for further review. This includes all cases of cardiac arrest or donor instability leading to loss of the donor prior to organ recovery. These events, with a narrative review, will be forwarded to the DSMC for review within 1 week. Incidence of all AEs (by study group) will be provided to the DSMC monthly and also be analyzed as part of the interim safety analysis. In addition, outcomes in the recipients receiving hearts from study participants will be analyzed in the interim analysis to ensure non-inferior graft function and survival.

### Frequency and plans for auditing trial conduct {23}

The study statistician (in consultation with the study coordinator and study PI) will audit data and conduct of the trial and oversee and provide feedback to each site coordinator. This will include monthly reviews of screening, enrolment, and data completion.

### Plans for communicating important protocol amendments to relevant parties (e.g., trial participants, ethical committees) {25}

Any important amendments to the protocol will be communicated to each study site within 1 week of the change. These will also be updated on clinicaltrials.gov.

## Dissemination plans {31a}

The final study results will be presented internally at the meeting of investigators. It will then be disseminated to the OPO community through the Organ Donation Research Council of AOPO and using the AOPO-plus Web portal. Further dissemination is planned through conference presentation(s) and journal publication(s).

## Discussion

This trial was discussed with at the AOPO councils of medical directors and of the Organ Donation Research Council. Feedback from these groups was incorporated into the study protocol. All OPOs were invited to participate and sent a survey to gauge interest.

## Trial status

Recruitment began on 01 December 2020. The anticipated date of study completion is 31 December 2022 (including 30-day recipient outcomes). The current protocol is version 3.0 (dated 01 November 2021).

## Supplementary Information

**Additional file 1.** Study Forms.

**Additional file 2.** Study Sites.

## Abbreviations

AOPO: Association of Organ Procurement Organizations
BD: Brain dead
DSMC: Data and safety monitoring committee
LVEF: Left ventricular ejection fraction
OPO: Organ procurement organization
ODRC: Organ Donation Research Council OPTN Organ Procurement and Transplant Network
SRTR: Scientific Registry of Transplant Recipients
T4: Thyroxine
TTE: Transthoracic echocardiography
UNOS: United Network for Organ Sharing
