# Supplementary material for: A multicenter randomized placebo-controlled trial of intravenous thyroxine for heart-eligible brain-dead organ donors
Source: Trials. 2021 Nov 27;22:852. doi: 10.1186/s13063-021-05797-2 (PMC8626969; doi:10.1186/s13063-021-05797-2)
Supplement: Supplementary file 1 — Additional file 1. Study Forms. [file 13063_2021_5797_MOESM1_ESM.docx]

**Title**

A Multicenter Randomized Placebo-Controlled Trial of Intravenous Thyroxine for Heart-Eligible Brain Dead Organ Donors

**NCT: 04415658**

**APPENDIX A: Study Forms**

**Document Date: 01 December 2020**

**A Multi-OPO Randomized Control Trial of T4 vs Placebo Screening Worksheet #1
**To be filled out on EVERY Brain-dead donor***

OPO: ________________________
Donor UNOS #: ________________
Date: ____ / ____ / 20____ (mm/dd/yyyy)
**Inclusion Criteria:**  (Must have *ALL 4* inclusion criteria)

1. _____ Declared dead by neurologic criteria
2. _____ Authorization for organ donation and research
3. _____ Donor age 14-55 years inclusive, and weight ≥ 45 kg (100 lbs.)
4. _____ Must be on 1 or more of the following vasopressors and/or inotropes at the start of the study:
   1. _____ norepinephrine
   2. _____ epinephrine
   3. _____ neosynephrine
   4. _____ dopamine
   5. _____ dobutamine
   6. _____ milrinone

**Exclusion Criteria:** (Must *NOT* have any exclusion criteria)

1. _____ Brain-death declared >24 hours ago
2. _____ CAD or MI (by hx, EKG, or previous cardiac cath) that would exclude transplantation
3. _____ Significant valvular heart disease (by hx or echo) that would exclude transplantation
4. _____ Previous sternotomy or cardiac surgery
5. _____ Donor is in a VA hospital
6. _____ Received IV or po T4/T3 in the last month (including home medication)
7. _____ Known HIV+ serology
8. _____ Other reason:_____________________________________________

*****If the donor has all 4 inclusion criteria and no exclusion criteria, he/she is eligible for the study and should be randomized to either T4 or NS, and the infusion should be started as soon as possible**.***

**ELIGIBLE** (go to worksheet) **NOT ELIGIBLE – STOP!**

All worksheets are to be submitted to the OPO research coordinator
Revised 10/22/2020

**Worksheet #2**

**Multi-OPO Randomized Control Trial of T4 vs Placebo Data Collection Worksheet**

UNOS number: __________________ (revised 11/5/2020)
OPO: _________________________________________________

1. Admission to the hospital: Date: ____ / ____ / 20____; Time: ____:____ (all times in 24:00)
2. Brain Death: Date: ____ /____ / 20____; Time: ____:____

Go to Sharepoint, **Multi-OPO T4 RCT randomization sheet** to identify the next randomization.

1. Randomized to: **T4** **NS**

***Draw ***free T4, TSH*** prior to starting T4 / NS infusion.

4a. Baseline results before starting T4 or saline: Weight _______ kg

4b. Vent settings: f _____ Vt _____ cc FiO2 _____ PEEP _____ PaO2 _____

Labs: (most recent before starting protocol) [enter 999 if data not available]

- 1. Highest Troponin I (before starting protocol) _________ ng/ml
  2. ALT _________
  3. AST _________
  4. Bilirubin __________
  5. Creatinine __________ BUN __________
  6. Amylase _________
  7. INR _________
  8. Calcium (total) _________ *or* k. Calcium (ionized) _________

1. T4 or NS started: Date: ____ /____ / 20____; Time: ____:____

***(needs to be started within 24 hours of brain-death declaration)***

1. T4 rate: 30mcg/hr [ 500 mcg/ 500 cc NS at 30 cc/hr = 30 mcg/hr]
   1. Was T4 started at 30 mcg/hr Yes ____ No ____
   2. If No, what was the reason: _______________________________________
2. NS rate 30 cc/hr [should be equal to the normal T4 dose, i.e. 30 cc/hr] *The donor may be on additional IV fluids as needed, but needs to have a separate IV of NS @ 30 cc/hr to match the fluid intake with the T4 protocol.*
   1. Was NS started at 30 cc/hr? Yes ____ No ____
   2. If No, what was the reason: ___________________________________________

**** Please fill out the Multi-OPO RCT T4 Vasopressor Flow Sheet #3. ****

*****The only allowed doses of T4 are 30 mcg/hr, 20 mcg/hr, 10 mcg/hr**. Decrease the dose by 10 mcg/hr, if a lower dose is needed.***

1. Was the T4 dose decreased before 12 hours? Yes ____ No ____

If Yes, what was the reason? (check all that apply)

1. ____Tachycardia (HR increased more than 20 bpm over baseline and is > 120 bpm)
2. ____Hypertension (Systolic BP increased >30 mmHg and is >180 mmHg)
3. ____Arrhythmia: ____ a fib/flutter; _____ SVT; _____ V tach; _____ PVCs(>6/min)
4. ____Other: __________________________________________________
5. Was the T4 dose stopped before 12 hours? Yes ____ No ____

If Yes, what was the reason? (check all that apply)

1. ____Tachycardia (HR increased more than 20 bpm over baseline and is > 120 bpm)
2. ____Hypertension (Systolic BP increased >30 mmHg and is >180 mmHg)
3. ____Arrhythmia: ____ a fib/flutter; _____ SVT; _____ V tach; _____ PVCs(>6/min)
4. ____Other: __________________________________________________
5. Time first echo ***ordered*** after T4/NS started: Date: ____ /____ / 20____; Time: ____:____
6. Ejection Fraction from first echo: __________%
7. If the donor was on T4 and completed the 12-hour protocol, was the T4 stopped within 60 minutes after the 12-hour study period? If the T4 stopped before 12 hours, answer N/A and go to #15.

_____ Yes [skip to #15] _____ No [go to #13] _____N/A [skip to #15]

If the donor was in the NS group, skip to question #14.

1. What was the reason the T4 was continued beyond the study period?
   1. Still on vasopressors ________
   2. Still on inotropes _________
   3. Physician’s preference ________­
   4. Other _____________________________________________
   5. Dose of T4 __________ mcg/hr
   6. Time T4 stopped: Date: ____ /____ / 20____; Time: ____:____

Go to Question #15.

1. If the donor was randomized to NS, did the donor receive T4 after enrollment in the study (“open-label”)?

____ Yes ____ No

If yes, what was the reason?

- 1. Still on vasopressors ________
  2. Still on inotropes _________
  3. Physician’s preference ________­
  4. Other _____________________________________________
  5. Dose of open-label T4 __________ mcg/hr
  6. Time open-label T4 started: Date: ____ /____ / 20____; Time: ____:____
  7. Time open-label T4 stopped: Date: ____ /____ / 20____; Time: ____:____

1. If more than 1 echo was performed, fill in the data below: [enter 999 in EF if not done]
   1. 2^nd^ echo: EF _______% Date: ____ /____ / 20____; Time: ____:____
   2. 3^rd^ echo: EF _______% Date: ____ /____ / 20____; Time: ____:____
   3. 4^th^ echo: EF _______ % Date: ____ /____ / 20____; Time: ____:____

**Draw *free T4* before going to the OR or at end of donor management on all donors in the study (T4 and control group)**

1. Baseline Serum free T4 _________ ng/dl TSH _________ mIU/L
2. Serum free T4 level _________ ng/dl prior to OR**.**
3. Was heart transplanted ? Yes______ No _________

If heart *not* transplanted, what was the reason? (check all that apply)

- 1. ____Decreased EF, poor function,
  2. ____CAD: 1 vessel ____ 2 vessel ____ 3 vessel ____
  3. ____LVH
  4. ____Size
  5. ____Medical history *(smoking, diabetes, HTN, etc)*
  6. ____Positive serologies
  7. ____HIV, HBV, HCV risk criteria
  8. ____Match list exhausted
  9. ____Recipient issues
  10. ____Intra-op decline
  11. ____Surgical injury
  12. ____Valvular disease
  13. ____Other __________________________________

**ADVERSE EVENTS**: Please identify **any new** adverse events that occurred in the donor, **regardless if they received T4 or NS**, after the start of the study:

1. **Did the donor experience any of the following adverse events?** Yes ____ No ____

If YES, please check all that occurred:

a.____ Severe hypertension (BP >200 mm Hg)

b.____ Tachycardia (HR> 150; including sinus tach, SVT, PSVT)

c.____ Fever (>102 degree)

d.____ A fib or a flutter

e.____ Ventricular ectopy (>6 VPCs/min, bigeminy, trigeminy)

f.____ V tach (spontaneously resolved)

g.____ V tach (requiring cardioversion)

h.____ V fib (requiring defibrillation)

i.____ cardiac arrest*

j.____ cardiac death prior to the OR*

k.____ new skin rash

l.____ other: ___________________________________________________

**19** m**. Was the adverse event related to the T4 infusion?**

____ YES ____ NO ____ UNCERTAIN

**19** n**. Please describe the adverse event:** _______________________________________

_________________________________________________________________________

*These events need to be reported to the DSMB.

20. Name of person filling out worksheet: ________________________________________

Date: ____________________________

***For the Research Coordinator:*** After the case is completed, please upload the SRTR data to the Excel Spread sheet.

## T4 Vasopressor Flow Sheet #3

**UNOS #: _____________________ OPO: _____________ Date Enrolled: _________ [***randomized to*  **T4: ___** *or* **NS: ___ ]**

|  |  |  |  |  | Blood Pressure | | | Vasopressor and inotrope doses (enter zero if not on the drug) | | | | | | |  |
| --- | --- | --- | --- | --- | --- | --- | --- | --- | --- | --- | --- | --- | --- | --- | --- |
| Interval | Time (Military)  (HR:min) | T4 Dose (mcg/hr) | Saline Dose  (cc/hr) | Heart Rate | - Invasive - Non-Invasive | | | Levophed*  (mcg/min) | Epi*  (mcg/kg/  min) | Neo*  (mcg/min) | Dopamine  (mcg/kg/  min) | Dobutrex*  (mcg/kg/  min) | Milrinone  (mcg/kg/  min) | Vaso*  (U/hr) | |
|  |  |  |  |  | Sys | Dias | |  |  |  |  |  |  |  |  |
| Baseline *Before* first dose of T4/NS | : |  |  |  |  |  | |  |  |  |  |  |  |  | |
| 2 Hours | : |  |  |  |  |  | |  |  |  |  |  |  |  | |
| 4 Hours | : |  |  |  |  |  | |  |  |  |  |  |  |  | |
| 6 Hours | : |  |  |  |  |  | |  |  |  |  |  |  |  | |
| 8 Hours | : |  |  |  |  |  | |  |  |  |  |  |  |  | |
| 10 Hours | : |  |  |  |  |  | |  |  |  |  |  |  |  | |
| 12 Hours | : |  |  |  |  |  | |  |  |  |  |  |  |  | |
| End of Donor MGMT1 |  |  |  |  |  |  | |  |  |  |  |  |  |  | |
| 12-hour intake: _____________ ml | | | |  | Date Vasopressor Ended | | | ___/___/___ | ___/___/___ | ___/___/___ | ___/___/___ | ___/___/___ | ___/___/___ |  | |
| 12-hour output:_____________ ml | | | |  | Time Vasopressor Ended | | | : | : | : | : | : | : |  |  |
| 12-hour NET I/O:_____________ ml | | | |  |  | |  | *Norepinephrine | *Epinephrine | *Neosynephrine |  | *Dobutamine |  | *Vasopressin | |

**Date/Time T4 stopped** *(Use Military Time):* _____/_____/_____ at _____:_____

**Notes/Comments: _____________________________________________________________________________________________________**

**Name of coordinator filling out worksheet: _______________________________________**

End of Donor Management is defined as when the donor was sent to the OR or was extubated in the ICU if no organs are procured.
