# Supplementary material for: A multicenter randomized placebo-controlled trial of intravenous thyroxine for heart-eligible brain-dead organ donors
Source: Trials. 2021 Nov 27;22:852. doi: 10.1186/s13063-021-05797-2 (PMC8626969; doi:10.1186/s13063-021-05797-2)
Supplement: Supplementary file 2 — Additional file 2. Study Sites. [file 13063_2021_5797_MOESM2_ESM.docx]

**Title**

A Multicenter Randomized Placebo-Controlled Trial of Intravenous Thyroxine for Heart-Eligible Brain Dead Organ Donors

**NCT: 04415658**

**APPENDIX B: Study Sites**

**Document Date: 01 October 2021**

|  | OPO code | OPO code |
| --- | --- | --- |
| 1 | Mid-America Transplant | MOMA |
| 2 | Southwest Transplant Alliance | TXSB |
| 3 | Texas Organ Sharing Alliance | TXSA |
| 4 | Lifesharing San Diego | CASD |
| 5 | Iowa Donor Network | IAOP |
| 6 | OurLegacy Florida | FLFH |
| 7 | Midwest Transplant Network | MWOB |
| 8 | Life Share of Oklahoma (inactive) | OKOP |
| 9 | Louisiana Organ Procurement Agency | LAOP |
| 10 | Donor Network of Arizona | AZOB |
| 11 | Donor Alliance | CORS |
| 12 | Lifebanc, Ohio | OHLB |
| 13 | LifeCenter Northwest (pending) | WALC |
| 14 | DonorConnect, Utah (pending) | UTOP |
